# The financing need of equitable provision of paid maternal leave in the informal sector in Indonesia: a comparison of estimation methods

**DOI:** 10.1186/s12939-021-01431-4

**Published:** 2021-04-06

**Authors:** Adiatma Y. M. Siregar, Pipit Pitriyan, Donny Hardiawan, Paul Zambrano, Roger Mathisen

**Affiliations:** 1 Center for Economics and Development Studies (CEDS), Department of Economics, Faculty of Economics and Business, Universitas Padjadjaran, Jl. Hayam Wuruk 6 – 8, Bandung, West Java 40115 Indonesia; 2 Center for Health Technology Assessment (CHTA), Universitas Padjadjaran, Bandung, West Java, Indonesia; 3 West Java Development Institute (INJABAR), Universitas Padjadjaran, Bandung, West Java, Indonesia; 4 Alive & Thrive, FHI 360, Southeast Asia, 7F, Opera Business Center, 60 Ly Thai To Street, Hanoi, Vietnam

**Keywords:** Informal sector, Breastfeeding, Maternal leave, Indonesia

## Abstract

**Background:**

Providing an enabling environment for breastfeeding is hampered by the inequitable implementation of paid maternity leave, primarily due to perceived or actual financial costs. To estimate the real cost of paid maternity leave requires using reliable methods. We compared methods utilized in two recent studies in Indonesia. Study A estimated the financial need of providing paid maternity leave in the formal sector with a 10-year forecast at 21% coverage of eligible mothers, while study B estimated similar costs for the informal sector at 100% coverage annually. Results are critical for guiding future application of either method to inform paid maternity leave policies.

**Methods:**

We compared number of covered mothers working informally, total annual cost, and cost per mother. We modified some parameters used in study A (method A) to be similar to study B (method B) for comparison, namely the period of estimate (annual), coverage (100%), estimate of women potentially breastfeeding, exchange rate, female labor force participation rate, the percentage of women working in the informal sector, and adding administration cost.

**Results:**

The methods differ in determining the number of mothers working in the informal sector who gave birth, the minimum wage as unit cost, and administrative cost. Both studies estimated the cost at various lengths of leave period. Method A requires more macro (e.g. national/regional) level data, while method B involves (e.g. individual) micro level data. We compared the results of method A with method B, respectively: 1) number of covered mothers working informally were 1,425,589 vs. 1,147,204; 2) total annual costs including administrative costs were US$650,230,167 vs. US$633,942,726, and; 3) cost/mother was US$456 vs US$553.

**Conclusion:**

Certain flexibilities can be applied to both methods, namely using parameters specific to respective regions (e.g. provincial level parameters), flexible period of analysis, and the use of administrative cost. In a setting where micro data is scarce and not easily accessible, method A provides a feasible approach, while method B will be most appropriate if suitable micro data is available. Future comparison studies in other settings are needed to provide further evidence on the strengths and weaknesses of both methods.

**Table:** 

This article is a part of the Interventions and policy approaches to promote equity in breastfeeding collection, guest-edited by Rafael Pérez-Escamilla, PhD and Mireya Vilar-Compte, PhD	

## Background

While the importance of breastfeeding has been recognized globally [1–5], reaching the World Health Assembly targets of at least 50 and 70% of infants exclusively breastfeeding up to 6 months (EBF) by 2025 and 2030, respectively [6] is not without challenges. Indonesia has an EBF rate of around 50% [7]. As Indonesia has the largest population among the ten member states of the Association of Southeast Asian Nations (ASEAN), it experiences the largest human and financial losses due to not breastfeeding [1, 3]. One of the persisting obstacles to women’s right to breastfeed is the limited and inequitable implementation of paid maternity leave [8, 9].

Indonesia has several relevant maternity protection policies enacted [10–17], although the effectiveness of their implementation still remains a crucial challenge [18, 19]. Policies mandating paid maternity leave in the informal sector are currently non-existent [19]. These are major gaps constraints since around 48% of approximately 70 million women of reproductive age (WRA) in Indonesia participate in the labor force, and of these, 52% work in the informal sector [20].

Implementing maternity protection is a form of social justice that supports women to better exercise their decision and protect their right to breastfeed [21, 22]. Maternity protection at the workplace “is a legal and social recognition of the contribution that women make by having babies while at the same time working for pay.” Thus, mothers are able to perform productive work and reproductive roles both at workplace and at home [23]. Paid maternity leave as a part of maternity protection is associated with better breastfeeding outcomes, provides broad social, developmental, and health benefits for working mothers and infants, promotes gender equity and may increase the female labor force participation rate. Such health benefits include averting the costs of sickness, cognitive losses and deaths due to not breastfeeding, and improve maternal-child physical and mental health and family wellbeing [1–5, 24–32]. Maternity cash transfer (MCT) is an intervention which eligible women receive a monthly unconditional cash transfer during pregnancy and through the child’s first 2 years of life (first 1000 days) [33], and can serve as an alternative to paid maternity leave for women working in the informal sector. Although the empirical evidence on the impact of MCT on breastfeeding outcomes is still limited, UNICEF suggests that social cash transfers can lead to higher EBF rates as the mother would be empowered to have more time for childcare [34, 35]. A few studies that have analysed the impact of cash transfers on education and health outcomes show that cash transfers may have a positive impact on breastfeeding [36–41].

One of the disincentives to implementing paid maternity leave is the perceived or actual financial costs to employers [19, 42, 43]. A reliable cost estimate of providing paid maternity leave is crucial for policy makers at the company and governmental level to inform policy decisions. Consequently, it is crucial to develop reliable methods to generate such estimates. A review has shown that of nine studies discussing the financing need of paid maternity leave [44], only two came from East Asia and Pacific region and one of them is from Indonesia [1, 19]. The study from Indonesia (study A) estimated the financial need of providing paid maternity leave in the formal sector for 10-years duration at 21% coverage of eligible women. Another upcoming study in Indonesia (study B) estimated similar costs to mothers working informally at 100% coverage annually [45]. Both studies share a relatively similar method with some key differences. To provide cost estimates of paid maternity leave, it is imperative to explore the available methods that have already incorporated the local context into its calculation.

This study aims to compare the methods utilized in the two studies to estimate the financial need of paid maternity leave in the formal and informal sectors in Indonesia. More specifically, we compared the methods of estimation, the number of covered mothers working informally, total annual cost, and unit cost per mother. Results are critical for guiding future application of either method to inform paid maternity leave policies and schemes in Indonesia. Globally, either methods’ framework can be useful in performing similar analysis given the specific data and policies available in respective countries.

## Methods

### Compared studies

This paper compared two studies published with data for Indonesia. Both studies aimed to estimate the financing need of paid maternity leave, although the sector discussed (i.e. formal vs. informal sectors) and methods used differ. Study A estimated the financing need of providing paid maternity leave in the formal sector with a ten-year forecast [19]. The number of women giving birth was estimated using the population aged 0–11 months from (adjusted) 2010 census data [46], while the percentage of mothers working formally was estimated using the National Labor Survey [47]. The coverage of eligible mothers was hypothetically increased gradually from 4 to 21% from the year 2020 to 2030 [8]. The female labor force participation rate [48] and the number of women giving birth also increased over time. The financing need is estimated for both three and 6 months paid leave. Study A also estimated the cost of setting up lactation rooms in up to 80% of medium and large companies within the same period.

Study B estimated the annual cost of providing a hypothetical monthly maternity cash transfer (MCT) for the informal sector at 21 and 100% coverage for 13, 14, 18, and 26 weeks after giving birth [45]. The study adapted the methods used in a previous study in Mexico [49]. The estimate included several types of unit costs, i.e. minimum wage, 2/3 minimum wage, unit cost of an existing conditional cash transfer program called *Program Keluarga Harapan* (PKH/Family Hope Program), and poverty line. Lastly, study B added administrative costs into its calculation.

### Methods adjustments

Table 1 presents the comparison of methods used in these studies, as well as the modifications made to the model on financing need of providing paid maternity leave in the formal sector (method A) in order to make its results comparable to the informal sector study (method B). We adjusted the former model into a one-year estimate at 100% coverage of women eligible for maternity leave (originally it was intended to estimate the financial need for 10 years at 21% coverage). We updated the estimate of population aged 0–11 months (as a proxy of women potentially breastfeeding) using National Socio Economic Survey (SUSENAS) 2018 data [20], adjusted the exchange rate and female labor force participation rate to be similar with study B, and multiplied these by the percentage of women working in the informal sector in method B. We did not alter the cost calculation itself to preserve the uniqueness of each method (e.g. method A used per province estimate of population aged 0–11 and minimum wages). We then compared the calculation methods and results, e.g. number of covered mothers working informally, total annual cost, and unit cost for each mother. These three results are the main findings of both papers and the most relevant information to policy makers.

**Table 1:** Assumptions and values used in the respective methods

Items	Method A [19]	Method B [44]	Modification made to method A or B for comparison
Type of intervention costed	Share of paid maternity leave payment between government and firms	MCT	MCT^+^
Exchange rate	Rp 13,120/US$ [50]	Rp 14,236/US$ [51]	Rp 14,236/US$^+^
Rate of cash benefit provided to employees by employers (%)	100 [8]	100 [8]	No changes
Period of estimation	10 years (estimated for 2020–2030)	Annual	Annual^+^
Unit cost(s)			
Minimum wage per month (US$)	110.6^	159.20 (39.80/week)*	Minimum wage per province were updated to 2018 values^+^
2/3 of minimum wage per month (US$)	–	106.13 (26.53/week)*	Excluded^++^
Family Hope cash transfer per month	–	168.59 (42.15/week) [52, 53]	Excluded^++^
Poverty line per month**	–	36.02 (9.01/week) [54, 55]	Excluded^++^
Percentage of working WRA	51.30 [48]	50.17 [20]	50.17^+^
Percentage of women working in formal/informal sector (out of working WRA	42.12 [56] (formal sector)	59.11 [20] (informal sector)	59.11 (informal sector) ^+^
Expected coverage of women in informal sector potentially eligible to receive paid maternity leave (%)	4.5 up to 21^a^ from 2020 to 2030 [8]	21^a^ and 100 [8] (one year, respectively)	Only 100 (one year) ^+, ++^
Total number of WRA working informally who gave birth covered (15–49 years)	1,687,364	1,147,204	1,425,589^+^
Length of maternity leave	3 and 6 months [57]	13, 14, 18 and 26 weeks [57, 58]	Only 3 months^+, ++^
Administration cost per female covered (US$)	–	35 (2018)*** [59]	35^+^

^+^Modification made to study A, ^++^ Modification made to study B, ^This is national level average wage, only serves to give a rough picture of the amount for the readers, the method itself used provincial minimum wage for its calculation [56]. The wage rate used for the calculation itself was the average wage rate per province; *the wage reflects average provincial minimum wage, compiled from various provincial regulation documents; **3.2US$ PPP 2011 per day, converted into 2018 nominal value using PPP conversion of Rp5,341.5/US$ and 2019 exchange rate, ***assumed to be similar to the Family Hope Program [59], adjusted to 2018 value using CPI of 147% (2010 = 100) [60]; ^a^Mean of coverage in law of maternity leave [8]

Table 1 shows the assumptions and values used in the calculation

## Results

Table 2 provides the steps followed in the adjusted method for comparison in this study. The first main difference in these two methods relates to how each determined the number of mothers who breastfeed. Method A used the population aged 0–11 months as the proxy, while method B used the number of females giving birth in the previous year. The second difference was in how each method determined the number of women working in the informal sector who gave birth. Method A multiplied the population aged 0–11 months per province by the national level female labor force participation rate and by the percentage of women working in the informal sector and summed the results. Method B used the proportion (termed α) of women working in the informal sector who gave birth in various subgroups out of the total women working informally and multiplied it by the national number of WRA working informally and summed the results. The third difference in these methods was how each determined the minimum wage as unit cost. Method A used different average minimum wage for respective provinces, while method B used a single average minimum wage rate. In this study, both methods estimated the financing need to provide maternity leave at 3 months.

**Table 2:** The steps of calculating annual costs of monthly MCT using the adjusted method for comparison

Step	Method A (adjusted)	Method B
1	Determine the number of the population between 0 and 11 months as a proxy of women who could be exclusively breastfeeding their infant (*Pop*_*p*_) per province.	Determine the number of women who work informally and gave birth in the prior year, given a vector of individual characteristics to form the number of WRA working in the informal sector in subgroups. Each subgroup presents the combination of WRA working in the informal sector based on several categories, namely aged (15–19, 20–24, 25–29, 30–34, 35–39, 40–44, 45–49), education (no education, primary education, junior high school, senior high school, diploma, and university), marital status (single, married, divorced, widow), locality (urban, rural), and gave birth in the last year (e.g. an example of a subgroup: the number of women working informally, aged 15–19, no education, single, live in urban area, gave birth in the last year).
2	Adjust the number of said population by parameters such as female labor force participation rate, share of women working in formal/informal sector, and the percentage of potential coverage of women who are eligible to receive paid maternity leave (*Adj*) to estimate the potential number of women who will receive paid maternity per province (*Pop*_*p*_ * *Adj*_*y*_)	Calculate the percentage of WRA working informally who gave birth in the prior year per subgroup as a share of the total WRA working informally (i.e. the number of WRA working informally who gave birth in the last one year in a subgroup/the total number of WRA working informally) to estimate α, defined as the probability of WRA working informally who gave birth in the last year within each of the subgroup.
3	Set the length of leave (*L*) to three months. Multiply the potential number of women who will receive paid maternity per province by length of leave (*Pop*_*p*_ * *Adj*_*y*_ * L)	Determine the beneficiaries who may claim maternity leave in the informal sector in a given year by weighting the population of WRA employed in the informal sector by α. *Pop*_*y*_ or WRA data at the population level were obtained from available data and adjusted by the percentage of female labor participation rate and adjusted further by the percentage of WRA who work informally. *Pop*_*y*_ was then multiplied by α of the respective subgroups to determine the number of WRA who works informally and gave birth within the prior year (α * Pop_*y*_*)*
4	Multiply the result from step three by the unit cost per province used to represent the value of cash transfer (*UC*_y_), e.g. average minimum wage per province (*Pop*_*p*_ * *Adj*_*y*_ * L * UC_y_)	Multiply the unit cost data (UC), e.g. average minimum wage, by results from step 3: (α * Pop_*y*_ * UC_*CT*_*)*
5	Add the proxy of administration cost to the calculation: (*Pop*_*p*_ * *Adj*_*y*_ * L * UC_y_) + *AdmCy*	Incremental coverage (IC) was determined based on regulations, recommendations, and literature regarding the length of leave and coverage. Multiply IC by step 4: (α * Pop_*y*_ * UC_*CT*_ * IC)
6	–	Add the proxy of administration cost to the calculation: (α * Pop_*y*_ * UC_*CT*_ * IC) + *AdmCy*

After adjusting the methods, we found the following results for method A and B, respectively: 1) the number of covered mothers working informally were 1,425,589 vs. 1,147,2049; 2) the total annual cost (with administration cost) was US$650,230,167 vs. US$633,942,726, and; 3) the unit cost per mother was US$456 vs US$553. Method A results in a greater number of informally working mothers covered and higher total annual cost, while having a lower cost per mother (Table 3). We cannot perform a mean difference t-test between results since we did not estimate the specific cost per mother and thus do not produce variabilities between costs.

**Table 3:** Results comparison

Items	Results		
	Method A	Method B	Note
Number of mothers working informally covered	1,425,589	1,147,204	278,385 difference, method B 20% lower
Total annual cost with administration cost	US$650,230,167	US$633,942,726	US$16,287,441 difference, method B 2.5% lower
Administration cost	US$50,192,140	US$40,390,767	US$9,801,373 difference, method B 20% lower
Unit cost per mother	US$456	US$553	US$96 difference, method A 17% lower

## Discussion

**Fig. 1:**
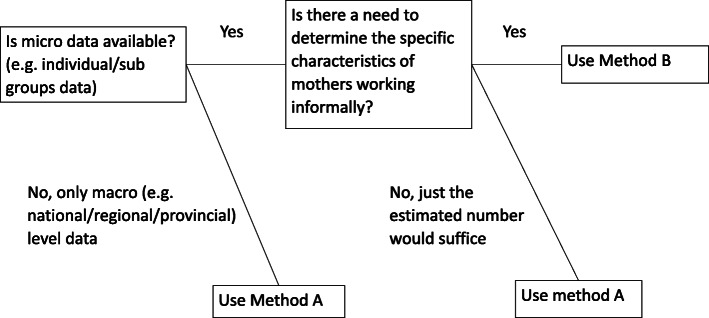
The potentially more practical decision of method given data availability. This figure shows the possible decisions to apply respective method

Methods A and B each have strengths and limitations, but both result in relatively similar estimates. Estimating mothers working informally who give birth, the decision of unit cost as a proxy to the value of paid maternity leave, and administrative costs are the key differences in both methods. As such, we argue that the decision on which method to use depends largely on data availability. Within the context of producing standardized maternity protection costing tool covering variations in data availability, maternity protection policies, the labor market, and fiscal structures in respective countries/regions, either methods can serve as costing model alternatives. Figure 1 proposes a framework to guide this decision. We discuss the following implications given methods differences in terms of performing analysis.

First, the estimate of mothers working informally who give birth is the primary difference in the methods used in respective studies. Method A used the number of populations aged 0–11 months per province and multiplied that number by the national level female labor force participation rate and percentage of women working in informal sector to produce the estimate. Such data are mostly accessible and, thus, the calculation can be easily performed. Although the results are relatively similar to those from method B, the use of macro data means that method A loses the variability in terms of individual/group characteristics receiving the MCT. Method B’s approach of first determining α, defined as the probability of WRA working informally who gave birth in the last year within each specific subgroup, captures this variability. As such, we may have more flexibility in calculating the cost of MCT that targets a certain group. However, the approach requires a certain data set that can capture such variability, which in some cases may not be readily accessible. In the case of Indonesia, the national socio-economic survey (SUSENAS) and national labor survey (SAKERNAS) are not freely available. As shown in Fig. 1, in a setting where micro data is scarce and not easily accessible, method A provides a feasible approach, while method B will be most appropriate to be used if suitable micro data is available.

Second, both methods have a certain range of flexibility which makes each highly adaptable to data availability. For instance, both methods can easily be modified to use parameters more specifically tailored to respective regions for even more accurate estimates (e.g. data of female labor force participation rate and percentage of mothers working informally at provincial level, if available). While method A is originally geared to estimate the financing need for longer than a one-year period, method B can also be modified to calculate longer periods by adding additional years to the calculation using varied parameters in correlation to the respective year of analysis. Similarly, the administrative cost used in method B can also be easily added to method A. People should be able to choose either method to perform similar estimates in line with the available data in their respective countries/regions as both methods offer a good level of flexibility while maintaining relatively similar level of accuracy. Furthermore, method A used provincial level data which could be built up to a national level analysis. This can be calculated beyond national level, to district/city, or municipal/village level data, depending on data availability. The micro level data will provide more regional variability, although it will still not capture the detailed individual/group characteristics variability found in method B. In this context, method B can also aggregate the analysis into province, district/city, municipal/village level, depending on the needs and data availability. Hence, the result can also be adjusted to national or lower administrative levels. Additionally, both methods can be used to calculate either the cost in formal and informal sectors as shown in this study for method A (modified to calculate the cost in informal sector), and in the study by Vilar-Compte et al [61] for method B (modified to calculate the cost in formal sector).

Third, similarly, the unit cost used in both methods can be flexible. For instance, in the case of Indonesia the minimum wage is also available for district/city level, thus resulting in 514 different values. These values can be applied to both methods A and B given that the data for the number of mothers working informally who give birth can support this level of analysis. This would give a more accurate cost estimate at the national level, although this would require much more extensive data which may not be available in all settings.

Methods A and B, as well as this study have a few limitations. First, methods A and B did not estimate other types of maternity benefit financing schemes, namely universal health coverage, social assistance benefits, and social insurance [62], thus the cost of maternity protection partially addressed by these other schemes is not recognized. Methods A and B provide only a (hypothetical) cash transfer scheme solely aimed at supporting maternity leave. However, even such information is currently lacking. Both methods have been able, to some extent, to show the required budget to support this important scheme.

Second, both methods did not address the important aspect of which level of governance pays for such a scheme (e.g. central or local government, or a combination of both). Originally, method A did address the potential cost share of government and employee, but still did not investigate the potential role of central and local government providing the budget. Further studies are needed to explore such a role and how it can be implemented within the current system.

Third, this study only focuses on Indonesia, while different countries will have their own data sets and local systems and, thus, the application and comparison of either method in other settings may result in different findings as compared to this study. However, we provided the initial framework to conduct similar studies in other settings, and future studies can perform such comparison and find out which method works better given their own data availability and local systems. This will enrich the currently limited studies of financing need for maternity leave, especially in the context of informal sector.

## Conclusion

Estimating the financing need to cover paid maternity leave or MCT requires methods that can be adjusted to available data. The methods discussed in this study can be easily modified to use parameters specifically tailored to respective countries or regions for accurate estimates (e.g. data on female labor force participation rate and percentage of mothers working informally at national or provincial level). As both methods result in relatively similar values, the determining factor would then be the data availability. If more extensive micro data is available, method B is preferable as it can capture more detailed population characteristics. In the absence of such data then method A is more feasible, although it may lose detailed population characteristics. Both methods present potential options to perform such calculations within different countries/regions, resulting in a richer and much needed, but currently lacking, database of the financing need to provide paid maternity leave. Ultimately, both estimation methods could contribute to a standardized maternity protection costing tool that is able to capture the variations of data and system in respective countries/regions.

## Data Availability

All calculations performed in this study is based on the studies by Siregar et al [19] and Vilar-compte et al [49]. As such all data and materials belong to the original authors.

## References

[1] Walters D, Horton S, Siregar AYM, Pitriyan P, Hajeebhoy N, Mathisen R, Phan LTH, Rudert C (2016). The cost of not breastfeeding in Southeast Asia. Health Policy Plan.

[2] Siregar AYM, Pitriyan P, Walters D (2018). The annual cost of not breastfeeding in Indonesia: the economic burden of treating diarrhea and respiratory disease among children (< 24mo) due to not breastfeeding according to recommendation. Int Breastfeed J.

[3] Walters DD, Phan LTH, Mathisen R (2019). The cost of not breastfeeding: global results from a new tool. Health Policy Plan.

[4] Victora CG, Bahl R, Barros AJD, França GVA, Horton S, Krasevec J, Murch S, Sankar MJ, Walker N, Rollins NC (2016). Breastfeeding in the 21st century: epidemiology, mechanisms, and lifelong effect. Lancet..

[5] Rollins NC, Bhandari N, Hajeebhoy N, Horton S, Lutter CK, Martines JC, Piwoz EG, Richter LM, Victora CG (2016). Why invest, and what it will take to improve breastfeeding practices?. Lancet..

[6] UNICEF, WHO. Global Breastfeeding Scorecard, 2019 (2019). Increasing Commitment to Brreastfeeding through Funding and Improved Policies and Programmes.

[7] Statistics Indonesia - Badan Pusat Statistik - BPS, National Population and Family Planning Board - BKKBN/Indonesia, Kementerian Kesehatan - Kemenkes - Ministry of Health/Indonesia, ICF International. Indonesia Demographic and Health Survey (IDHS) 2017. 2018;1–606.

[8] International Labour Organization (ILO) (2014). Maternity and paternity at work. Law and practice across the world.

[9] Smith JP, Javanparast S, Mcintyre E, Craig L, Mortensen K, Koh C (2013). Discrimination against breastfeeding mothers in childcare. Aust J Labour Econ.

[10] Ministry of Manpower and Transmigration (2003). Manpower Law No. 13/2003.

[11] The President of Republic of Indonesia (2012). Government Regulations No. 33/2012.

[12] Ministry of Women Empowerment and Child Protection, Ministry of Labor, Ministry of Health (2008). Agreement between three Ministries.

[13] The President of Republic of Indonesia (2009). Government Regulation on Health No. 36/2009.

[14] Ministry of Health of the Republic Indonesia (2014). Ministry of Health Regulation No. 15 Year 2014.

[15] Ministry of Health of the Republic of Indonesia (2013). Ministry of Health Regulation no. 39 Year 2013.

[16] Ministry of Health of the Republic of Indonesia (1994). Ministry of Health Decree No. 386/MEN.KES/SK/IV/1994 - Annex.

[17] Ministry of Health of the Republic Indonesia (2014). Ministry of Health Regulation No. 49 Year 2014.

[18] Shetty P (2014). Indonesia’s breastfeeding challenge is echoed the world over. Bull World Health Organ.

[19] Siregar AYM, Pitriyan P, Walters D, Brown M, Phan LTH, Mathisen R (2019). The financing need for expanded maternity protection in Indonesia. Int Breastfeed J.

[20] National Bureau of Statistics (BPS) (2018). Indonesia - National Social Economic Survey (SUSENAS) 2018.

[21] Núñez AE (2016). Good intention is not enough: intentional action to address health disparities in breastfeeding. Breastfeed Med.

[22] Boccolini CS, Pérez-Escamilla R, Giugliani ERJ, de MM BP (2015). Inequities in Milk-Based Prelacteal Feedings in Latin America and the Caribbean. J Hum Lact.

[23] Harooni N, Petitat-Cote E, Arendt M, de Maza V (2015). Maternity protection at the workplace.

[24] Heymann J, Sprague AR, Nandi A, Earle A, Batra P, Schickedanz A (2017). Paid parental leave and family wellbeing in the sustainable development era. Public Health Rev.

[25] Hajizadeh M, Heymann J, Strumpf E, Harper S, Nandi A (2015). Paid maternity leave and childhood vaccination uptake: longitudinal evidence from 20 low-and-middle-income countries. Soc Sci Med.

[26] International Labour Organization (ILO). Cash transfer programmes, poverty reduction and empowerment of women: a comparative analysis. Geneva; 2013.

[27] Van Niel MS, Bhatia R, Riano NS, de Faria L, Catapano-Friedman L, Ravven S (2020). The impact of paid maternity leave on the mental and physical health of mothers and children: a review of the literature and policy implications. Harv Rev Psychiatry.

[28] Mirkovic KR, Perrine CG, Scanlon KS (2016). Paid maternity leave and breastfeeding outcomes. Birth..

[29] Jia N, Dong X, Song Y (2018). Paid maternity leave and breastfeeding in urban China. Fem Econ.

[30] Sinha B, Chowdhury R, Sankar MJ, Martines J, Taneja S, Mazumder S, Rollins N, Bahl R, Bhandari N (2015). Interventions to improve breastfeeding outcomes: a systematic review and meta-analysis. Acta Paediatr Int J Paediatr.

[31] Chai Y, Nandi A, Heymann J (2018). Does extending the duration of legislated paid maternity leave improve breastfeeding practices? Evidence from 38 low-income and middle-income countries. BMJ Glob Health.

[32] Bonet M, Marchand L, Kaminski M, Fohran A, Betoko A, Charles MA (2013). Breastfeeding duration, social and occupational characteristics of mothers in the French “EDEN mother-child” cohort. Matern Child Health J.

[33] Field E, Maffioli E (2019). The impact of maternal and child cash transfers on malnutrition.

[34] UNICEF-ESARO/Transfer Project (2015). Social Cash Transfers and Children’s Outcomes: A review of evidence from Africa.

[35] Groot R de, Palermo T, Handa S, Ragno LP, Peterman A. Cash transfers and child nutrition: what we know and what we need to know. Florence: UNICEF Office of Research; 2015.

[36] Relton C, Strong M, Thomas KJ, Whelan B, Walters SJ, Burrows J, Scott E, Viksveen P, Johnson M, Baston H, Fox-Rushby J, Anokye N, Umney D, Renfrew MJ (2018). Effect of financial incentives on breastfeeding: a cluster randomized clinical trial. JAMA Pediatr.

[37] Anokye N, Coyle K, Relton C, Walters S, Strong M, Fox-Rushby J. Cost-effectiveness of offering an area-level financial incentive on breast feeding: a within-cluster randomised controlled trial analysis. Arch Dis Child. 2020;105:155–9. 10.1136/archdischild-2018-316741 PMC7025724 31444210

[38] Whelan B, Relton C, Johnson M, Strong M, Thomas KJ, Umney D, Renfrew M (2018). Valuing breastfeeding: health care professionals’ experiences of delivering a conditional cash transfer scheme for breastfeeding in areas with low breastfeeding rates. SAGE Open.

[39] Becker F, Anokye N, de Bekker-Grob EW, Higgins A, Relton C, Strong M, Fox-Rushby J (2018). Women’s preferences for alternative financial incentive schemes for breastfeeding: a discrete choice experiment. PLoS One.

[40] Powell-Jackson T, Mazumdar S, Mills A (2015). Financial incentives in health: new evidence from India’s Janani Suraksha Yojana. J Health Econ.

[41] Renzaho AMN, Chen W, Rijal S, Dahal P, Chikazaza IR, Dhakal T, et al. The impact of unconditional child cash grant on child malnutrition and its immediate and underlying causes in five districts of the Karnali zone, Nepal - a trend analysis. Arch Public Heal. 2019;77. 10.1186/s13690-019-0352-2 PMC6540561 31161038

[42] Lewis S, Stumbitz B, Miles L, Rouse J (2014). Maternity protection in SMEs - an International Review.

[43] Stumbitz B, Leal Filho W, Azul AM, Brandli L, Lange Salvia A, Wall T (2020). Maternity protection at work: decent work for all during pregnancy and new motherhood. Decent work and economic growth.

[44] Carroll G, Safon C, Buccini G, Vilar-Compte M, Teruel G, Pérez-Escamilla R. A systematic review of costing studies for implementing and scaling-up breastfeeding interventions: what do we know and what are the gaps? Health Policy Plan. 2020;35:461–501. 10.1093/heapol/czaa005 32073628

[45] Siregar AYM, Pitriyan P, Hardiawan D, Zambrano P, Vilar-Compte M, Belismelis GMT, et al. The yearly financing need of providing paid maternity leave in the informal sector in Indonesia. Int Breastfeed J. 2021;16:17. 10.1186/s13006-021-00363-7 PMC7885595 33588917

[46] Badan Pusat Statistik (BPS) - Central Bureau Statistics (2011). Population Census 2010.

[47] Directorate of Demography and Labor Statistics (2019). National Labor Survey 2018.

[48] World Bank (2016). Labor force participation rate, female (% of female population ages 15+), Modeled ILO Estimate.

[49] Vilar-Compte M, Teruel G, Flores D, Carroll GJ, Buccini GS, Pérez-Escamilla R (2019). Costing a maternity leave cash transfer to support breastfeeding among informally employed Mexican women. Food Nutr Bull.

[50] Bank of Indonesia (2016). Reference Exchange Rate - Jakarta Interbank Spot Dollar Rate (JISDOR), USD - IDR.

[51] Bank of Indonesia. Foreign Exchange Rates. Bank of Indonesia. 2019. https://www.bi.go.id/en/moneter/informasi-kurs/transaksi-bi/Default.aspx. Accessed 24 Jun 2019.

[52] Minister of Social Affair (2018). Ministry of Social Affair Regulation no. 1 year 2018.

[53] Ministry of Social Affair (2019). The distribution of social assistance, Family Hope Program 2019.

[54] The World Bank (2019). Indonesia. Poverty Equity Br East Asia Pacific.

[55] Azka RM. Do not measure poverty using exchange rate. Bisnis.com. 2018.

[56] Directorate of Demography and Labor Statistics (2013). National Labor Survey 2012. Badan Pusat Statistik (BPS) - Central Bureau Statistics.

[57] Ministry of Manpower and Transmigration (2003). Labor Decree no. 13, Article 93.

[58] WHO/UNICEF (2014). Global nutrition targets 2025: breastfeeding policy brief (WHO/NMH/NHD/14.7).

[59] The World Bank (2012). Program Keluarga Harapan (PKH) conditional cash transfer.

[60] The World Bank (2019). Consumer price index (2010 = 100).

[61] Vilar-Compte M, Teruel GM, Flores-Peregrina D, Carroll GJ, Buccini GS, Perez-Escamilla R (2020). Costs of maternity leave to support breastfeeding; Brazil, Ghana and Mexico. Bull World Health Organ.

[62] International Labour Organization (2012). Maternity Protection Resource Package - From Aspiration to Reality for All. Module 6: Maternity leave and related types of leave.

